# 675-nm diode laser as an adjuvant treatment for telogen effluvium: case series

**DOI:** 10.3389/fmed.2026.1753577

**Published:** 2026-04-22

**Authors:** Angelica Ruiz Dueñas, Luis Enrique Sanchez Dueñas, Miroslaba Rodríguez Puente, Nicole Orendain Koch

**Affiliations:** 1 Private Practitioners, Zapopan, Mexico; 2 Dermika Centro Dermatológíco Láser, Guadalajara, Mexico

**Keywords:** 675-nm diode laser, alopecia, diode laser, photobiomodulation therapy, telogen effluvium

## Abstract

**Background:**

Telogen effluvium (TE) is a diffuse, non-scarring alopecia that can have a significant impact on quality of life. Although often self-limited, many patients seek therapies that speed recovery. Photobiomodulatory benefits of the 675-nm diode laser are also well established in treating other hair disorders, but there is limited evidence in TE.

**Methods:**

We performed a retrospective case series of 13 patients diagnosed with TE at a Dermatologic Laser Center in Mexico. The patients received adjuvant treatment with 675-nm diode laser therapy.

**Results:**

Of the 13 patients, 69% had chronic TE. Among those with a positive baseline pull test, 72% became negative at follow-up. Pruritus improved in 83% of symptomatic patients, and trichodynia resolved in 75%. No adverse events occurred.

**Conclusion:**

675-nm diode laser seems to be a safe, well-tolerated, and potentially effective adjuvant therapy for TE. Controlled studies are needed to confirm efficacy and establish standardized protocols.

## Introduction

Telogen effluvium (TE) is defined by premature shift of hair follicles toward the telogen phase, which causes diffuse shedding that can significantly impact patient quality of life. While TE typically resolves spontaneously after removal of the trigger, the length of time required to recover often drives patients to pursue supportive adjuvant therapies. Photobiomodulation (PBM) using low-level light or laser devices has shown potential benefits in hair disorders; but data for TE are still limited. This case series is unique cause it describes for the first time to our knowledge, the use of a 675-nm diode laser as an adjuvant modality in TE management, contributing new observational evidence to the medical literature.

## Case series

We present a retrospective case series of 13 telogen effluvium patients treated with 675-nm diode laser as an adjuvant therapy. Patients were treated by trichologists at the Hair Restoration Center of Dermika Centro Dermatológico Láser in Guadalajara, Jalisco, Mexico. The follow-up period ranged from 2 to 3 months, depending on each physician’s criteria. The diagnosis of TE was established based on clinical history, trichoscopy, and the pull test. Of the 13 patients included, 3 were male (23%) and 10 were female (77%). The average age was 46.6 years; 9 patients had chronic TE (69%) and 4 had acute TE (31%). Among those with chronic TE, the average duration of evolution was 49 months, whereas for acute TE the duration was 3.5 months. Identified triggering factors included stress in 4 patients (30%), weight loss in 3 patients (23%), and a history of dengue fever in 2 patients (15%). Most had positive pull test (85%); 6 patients reported scalp pruritus (46%) and 4 reported trichodynia (30%). Comorbidities reported included androgenetic alopecia in 11 patients (84%), vitamin D insufficiency in 2 (15%), and thyroid disease in 2 (15%) (Table 1).

**Table 1:** Baseline characteristics of patients with telogen effluvium (*n* = 13).

ID	Sex	Age (years)	Telogen effluvium	Duration (months)	Triggering factors	Pull test	Pruritus	Trichodynia	Comorbidities
1	M	28	Chronic	36	Stress	Positive	No	No	Androgenetic alopecia
2	F	35	Acute	1	Weight loss	Positive	No	No	Polycystic ovary syndrome, endometriosis
3	F	35	Chronic	24	Not identified	Positive	Yes	No	Vitamin D insufficiency
4	F	56	Chronic	24	Not identified	Positive	Yes	No	Frontal fibrosing alopecia, androgenetic alopecia
5	F	54	Chronic	24	Menopause, weight loss, stress	Positive	Yes	Yes	Androgenetic alopecia, insulin resistance, hypercholesterolemia
6	F	21	Chronic	72	Stress, hair extensions, anemia, dengue	Negative	Yes	Yes	Androgenetic alopecia, anemia
7	F	59	Acute	6	Weight loss, hypothyroidism	Positive	No	No	Androgenetic alopecia, seborrheic dermatitis, hypothyroidism, autoimmune disease
8	F	57	Chronic	9	Not identified	Negative	No	No	Androgenetic alopecia, seborrheic dermatitis, vitamin D insufficiency
9	F	63	Acute	4	Stress	Positive	Yes	Yes	Fibromyalgia, hyperthyroidism
10	F	59	Chronic	120	Dengue and hormone replacement therapy	Positive	No	No	Androgenetic alopecia
11	M	32	Acute	3	COVID vaccine	Positive	Yes	Yes	Androgenetic alopecia
12	M	32	Chronic	12	Not identified	Positive	No	No	Androgenetic alopecia
13	F	75	Chronic	120	Not identified	Positive	No	No	Androgenetic alopecia, alopecia areata, hypothyroidism, venous insufficiency, gout

Chronic telogen effluvium was defined as hair shedding lasting longer than 6 months; acute telogen effluvium was defined as shedding lasting ≤6 months.

All patients received adjuvant treatment with 675-nm diode laser (RedTouch DEKA) using the following parameters: 6 J/cm^2^/0.5 W, macro 13 mm/micro 0.7 mm, standard-pulsed mode (25 ms), one pass per area, with a total duration of 20 min per session. The number of sessions was determined by the patient’s financial means, as the treatment is costly. In general, we recommend a minimum of three sessions. Most patients completed a total of 3 sessions, with 4-week intervals between each. The main treatment remained unchanged throughout the course of therapy depending on individual needs, and the overall adherence rate was 79% (Table 2).

**Table 2:** Treatment parameters, adherence, and clinical outcomes of patients receiving 675-nm diode laser therapy.

ID	Sessions completed	Interval between sessions (weeks)	Co-treatments	Adherence to treatment (%)	Pull test	Pruritus	Trichodynia	Adverse effects
1	3	4	Minoxidil 5 mg/day, dutasteride 0.5 mg/day	100	Negative	No	No	No
2	3	4	Minoxidil 5 mg/day, proteoglycans	100	Negative	No	No	No
3	2	4	Minoxidil lotion 5%	80	Positive	No	No	No
4	3	4	Minoxidil 2.5 mg/day, dutasteride 0.5 mg/day	100	Negative	No	No	No
5	3	4	Minoxidil 2.5 mg/day, dutasteride 0.5 mg 3x week, proteoglycans, multivitamin, vitamin D 4,000 UI/day, minoxidil lotion 5%	80	Negative	No	No	No
6	1	4	Vitamin D 4000 UI/day, iron 100 mg-folic acid 800 mcg/day, minoxidil 0.5 mg/day, dutasteride 0.5 mg 3x week, proteoglycans, clobetasol lotion 0.05% 1 month	50	Negative	No	No	No
7	1	4	Multivitamin, minoxidil 0.25 mg/day, clobetasol lotion 0.05%, minoxidil foam 5%	20	Negative	Yes	No	No
8	3	6	Anti hair loss lotion	30	Negative	No	No	No
9	3	4	Dutasteride 0.5 mg/day, minoxidil 1 mg/day, clobetasol lotion 0.05% 1 month	70	Negative	No	No	No
10	3	12	Mesotherapy (niacinamide, acetylcysteine, biotin)	100	Positive	No	No	No
11	4	4–13	Mesotherapy (niacinamide, acetylcysteine, biotin, dutasteride 0.01%)	100	Positive	Yes	Yes	No
12	9	0.5–1	Mesotherapy (exosomes)	100	Negative	No	No	No
13	18	1–4	Mesotheraphy (exosomes)	100	Negative	No	no	No

Laser therapy was performed with a 675-nm diode laser at 6 J/cm^2^, 0.5 W, pulsed mode (25 ms), macro 13 mm/micro 0.7 mm, with one pass per area and a total duration of 20 min per session.

**Figure 1:**
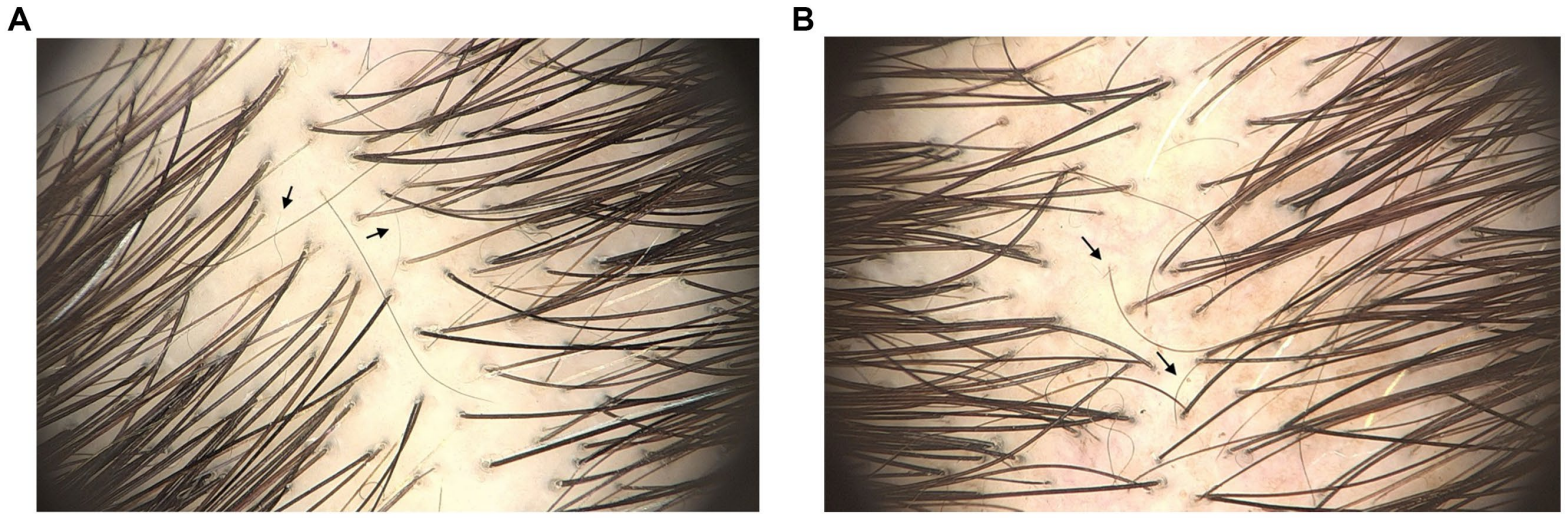
**(A)** Baseline trichoscopic image of a patient with chronic telogen effluvium (FotoFinder dermoscope, ×20 magnification). Decreased hair density and numerous short regrowing hairs (arrows) are observed, without significant follicular miniaturization. **(B)** Follow-up trichoscopic image obtained 1 year after treatment (FotoFinder dermoscope, ×20 magnification). Increased hair density and regrowing hairs (arrows) are observed, with preserved hair shaft diameter uniformity.

At follow-up, out of 11 patients with a positive pull test at initial evaluation, 8 tested negative (72%). None of the patients with a negative pull test became positive. Of the 6 patients who reported pruritus at baseline, symptoms resolved in 5 (83%), and 3 patients with initial trichodynia experienced complete resolution (75%). Trichoscopic improvement was also observed, as illustrated in Figure 1, with increased hair density, reduced empty follicles, and a higher proportion of thicker terminal hairs. No adverse effects were reported in any patient (Table 2).

## Discussion

The present case series provides preliminary observational evidence supporting the potential utility of 675-nm diode laser as an adjuvant therapy in the management of TE. Although TE is traditionally considered a self-limited disorder when the cause is treated, the clinical burden experienced by affected individuals—including psychological distress, impaired quality of life, and prolonged recovery—creates an increasing need for non-invasive therapeutics capable of accelerating restoration of normal hair cycling. However, until now, published data have focused almost exclusively on androgenetic alopecia (AGA).

The biological plausibility for using 675-nm laser technology in TE derives from its unique photophysical profile and subsequent photobiomodulatory effects. The wavelength of 675 nm exhibits selective affinity for dermal chromophores—particularly collagen and melanin—while minimizing interaction with hemoglobin and water, thus enabling targeted energy deposition into the follicular microenvironment with minimal thermal injury (1, 2). Preclinical studies have shown that irradiation within this red-light spectrum enhances mitochondrial cytochrome c oxidase activity, increases adenosine triphosphate (ATP) production, strengthens antioxidant pathways, and modulates transcription factors that govern cellular proliferation and inflammatory signaling (3–5). These pathways are also relevant for TE, where dysfunctional cycling, oxidative stress, and transient microinflammation contribute to follicular instability. By promoting re-entry into anagen and supporting follicular metabolic activity, 675-nm laser could in principle counteract persisting shedding and promote recovery.

Clinical evidence supporting 675-nm applications in hair disorders primarily originates from AGA research. Sorbellini et al. reported that a 10-session protocol of 675-nm laser therapy resulted in significant increases in hair density, reduction in miniaturization markers, and favorable dermoscopic changes without adverse events (6). Similarly, Tolone et al. reviewed current 675-nm clinical applications and emphasized its consistent safety profile and ability to stimulate type III collagen synthesis while preserving keratinocyte viability (2). While these studies focus on AGA, the fundamental biological properties—promotion of anagen, modulation of perifollicular inflammation, and improvement of dermal architecture—are also applicable to TE. Because TE lacks the structural follicular damage observed in AGA, follicles are theoretically more responsive and capable of rapid functional restoration under photobiomodulatory stimulation.

Importantly, the majority of patients who initially presented with a positive pull test achieved normalization by follow-up, consistent with improved anchoring of anagen-transitioning hairs. In addition, symptomatic improvements of pruritus and trichodynia, leading signs of subclinical neuroinflammatory function, implies a potential modulation of sensory nerve fibers and inflammatory mediators consistent with previous PBM studies, which have demonstrated a reduction in nitric oxide availability and modulation of inflammatory cytokines (7, 8). The observed absence of adverse events reinforces the safety profile reported in prior 675-nm dermatologic investigations across melasma, acne scarring, and photoaging (2, 9–11).

Another relevant observation is the high prevalence of AGA comorbidity within our case series. Mixed TE-AGA presentations are prevalent in clinical practice and often complicate diagnosis and management. In such cases, TE can unmask or exacerbate underlying pattern hair loss, which makes treatments targeting both treatment pathways immensely helpful. Given that 675-nm laser therapy has demonstrated efficacy in early-stage AGA (6), its dual applicability in mixed-etiology hair shedding represents a practical advantage over traditional TE-focused approaches, which rely heavily on trigger identification and supportive care without directly enhancing follicular activity. The reduction in shedding and symptom improvement observed in this case series may reflect both resolution of TE stimuli and early regenerative effects on AGA-susceptible follicles.

Although results were encouraging, we must note several limitations. First, the lack of quantitative trichoscopic metrics (e.g., hair density counts, anagen-telogen ratios, shaft diameter analysis) restrict objective evaluations of the treatment impact. While symptom resolution and pull-test normalization are clinically meaningful in TE, standardized measurement tools should be included in future studies to confirm morphological improvement. Second, the small sample size and lack of a control group limit the ability to identify whether treatment effects deviate from the natural course of TE, especially for acute types of cases, when spontaneous improvement can be expected in the first few months. Randomized controlled trials comparing 675-nm laser therapy versus sham treatment or versus established PBM modalities (e.g., 650–655 nm LLLT devices) would allow for meaningful comparison and determine whether the wavelength-specific properties of 675 nm confer superior benefits. Third, the heterogeneity of comorbidities and co-treatments introduces potential confounders. Many patients received supplements or pharmacologic therapy for associated conditions such as AGA, vitamin D insufficiency or thyroid dysfunction—interventions that may influence hair shedding. Standardizing treatment protocols and stratifying analyses by comorbidity profiles would improve interpretability. Additionally, while adherence to treatment averaged 79%, determining whether outcomes differ between high- and low-adherence subgroups would help elucidate dose–response characteristics.

However, the lack of any adverse effect and the positive patient-reported outcomes suggest that this approach is clinically feasible. The tolerability benefit is significantly greater compared to pharmacologic agents, such as minoxidil or dutasteride, which may be contraindicated, poorly tolerated, or undesired by patients. For TE patients with increased anxieties about continued shedding, the quick symptomatic relief of PBM might provide psychological benefit in addition to physiologic success. Further studies should focus on those questions which remain unanswered (best treatment periods, sessions needed, long-lasting durability in improvement and biological markers of response). Moreover, investigating combination approaches—such as integrating 675-nm therapy with microneedling, platelet-rich plasma, or topical agents—may reveal synergistic effects, as observed in other hair-loss conditions (12–14).

Taken together, the findings of this case series, in conjunction with existing literature, support the rationale for further exploration of 675-nm laser therapy in TE. While evidence remains preliminary, the observed symptomatic improvements, normalization of pull test results, and consistent safety profile position this wavelength as a promising addition to the therapeutic armamentarium for TE—particularly in patients with refractory symptoms, mixed TE-AGA presentations, or preference for non-pharmacologic interventions.

## Conclusion

This case series suggests that 675-nm diode laser PBM may represent a safe, well-tolerated, and potentially effective adjuvant therapy for telogen effluvium. The biological mechanisms of this wavelength, coupled with prior evidence in androgenetic alopecia and other dermatologic conditions, support its role in promoting follicular recovery and reducing symptoms. Although additional controlled studies with standardized outcomes are required to confirm efficacy, the present findings contribute meaningful preliminary evidence that 675-nm laser therapy may accelerate clinical improvement in TE and enhance overall patient satisfaction.

## Data Availability

The original contributions presented in the study are included in the article/supplementary material, further inquiries can be directed to the corresponding author.
